# Parent‐Specific Transgenerational Immune Priming Enhances Offspring Defense—Unless Heat Stress Negates It All

**DOI:** 10.1002/ece3.70552

**Published:** 2024-11-25

**Authors:** Ralf F. Schneider, Arseny Dubin, Silke‐Mareike Marten, Olivia Roth

**Affiliations:** ^1^ Department of Zoology, Marine Evolutionary Biology University of Kiel Kiel Germany; ^2^ Department of Marine Evolutionary Ecology Helmholtz Centre for Ocean Research Kiel Germany

**Keywords:** broad‐nosed pipefish, ecology, microbiome, *Syngnathus typhle*, trained immunity, transcriptome

## Abstract

Transgenerational immune priming (TGIP) adjusts offspring's immune responses based on parental immunological experiences. It is predicted to be adaptive when parent–offspring environmental conditions match, while mismatches negate those advantages, rendering TGIP potentially costly. We tested these cost–benefit dynamics in the pipefish *Syngnathus typhle* (Syngnathidae). Because of their unique male pregnancy, egg production and rearing occur in different sexes, providing both parents multiple avenues for TGIP. Parental bacteria exposure in our pipefish was simulated through vaccinations with heat‐killed *Vibrio aestuarianus* before mating the fish to each other or to controls. The resulting offspring were exposed to *V. aestuarianus* in control or heat stress environments, after which transcriptome and microbiome compositions were investigated. Transcriptomic TGIP effects were only observed in *Vibrio*‐exposed offspring at control temperatures, arguing for low costs of TGIP in non‐matching microbiota environments. Transcriptomic phenotypes elicited by maternal and paternal TGIP had limited overlap and were not additive. Parentally induced transcriptomic responses were associated with immune functions, and specifically, the paternal response to the innate immune branch, possibly hinting at trained immunity. TGIP of both parents reduced the relative abundance of the experimental *Vibrio* in exposed offspring, showcasing its ecological benefits. Despite TGIP's significance in matching biotic environments, no TGIP‐associated phenotypes were observed for heat‐treated offspring, illustrating its limitations. Heat spikes caused by climate change thus threaten TGIP benefits, potentially increasing susceptibility to emerging marine diseases. We demonstrate the urgent need to understand how animals cope with climate‐induced changes in microbial assemblages to assess their vulnerability in light of climate change.

## Graphical abstract

Transgenerational immune priming allows parents to prepare their offspring for the microbial environment they will be released into. Using transcriptomics and microbiome analyses, we show that the male pregnant pipefish *Syngnathus typhle* primes its offspring after parental vaccination with Vibrio bacteria, that priming is effective in reducing Vibrio abundance after a contact with the offspring after release, and that this priming is Vibrio‐specific and not costly if the specific Vibrio is not encountered by the offspring. We also show that this mechanism is losing its effectiveness under heat stress conditions, arguing for the threat heat waves pose for inhabitants of shallow coastal habitats, such as the seagrass meadows inhabited by *S. typhle*.

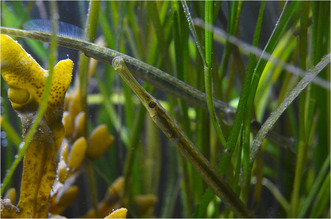

## 1 Introduction

Transgenerational plasticity provides an evolutionary opportunity for short‐term acclimatization to changing environmental conditions by shaping the offspring phenotype through the transfer of parental experience (Fox and Mousseau 1996; Kirkpatrick and Lande 1989; Rossiter 1996; Roth et al. 2018). Transgenerational plasticity can be adaptive and improve offspring survival in situations of rapid but predictable changes in abiotic conditions [e.g., temperature and salinity in marine habitats (Bautista and Crespel 2021; Massamba‐N'Siala, Prevedelli, and Simonini 2014; Serobyan and Sommer 2017; Shama et al. 2014; Ye et al. 2019)] as well as biotic conditions (e.g., changing microbial assemblage) in a matching parent–offspring environment (Roth et al. 2018; Schneider and Meyer 2017; Tetreau et al. 2019). In the latter situation, transgenerational immune priming (TGIP; the transfer of parental immunological experience) was suggested to enhance offspring immune responses toward an infectious agent that parents have previously encountered. It encompasses a changed epigenetic programming and/or a direct transfer of parental substances (Tate and Van Cleve 2022), including cells, cytokines, and antibodies (Jennewein et al. 2017) that together shape neonatal immunity (Albrecht and Arck 2020; Niewiesk 2014). Our recently broadened understanding of TGIP mechanisms (Heckwolf et al. 2020; Pappert et al. 2023; Roth et al. 2018; Tanger et al. 2023), in particular, the roles assigned to epigenetic programming as well as the microbiome, has also expanded our understanding of potential routes of TGIP. While traditionally regarded as a maternal trait via deposition into the maternal eggs (Hasselquist and Nilsson 2009; Sadd et al. 2005) or provisioning through maternal care [pregnancy or post‐natal feeding (Laouar 2020; Liu, Zhong, and Zhang 2022; Nyangahu and Jaspan 2019; Stelzer et al. 2021)], the father's role in TGIP has recently begun to be explored (Baldassarre et al. 2021; Beemelmanns and Roth 2016a, 2016b; Debnath and Berk 2023; Keightley, Wong, and Lieschke 2013; Roth et al. 2010; Roth, Klein, et al. 2012; Rutkowski et al. 2023; Sternberg, de Roode, and Hunter 2015). To this end, TGIP entails a plethora of distinct routes on how immunological experience of the innate or adaptive immune branch can be transferred into the next generation to support the onset of an effective immunological defense in early life (Albrecht et al. 2022; Frolows and Ashe 2021; Netea et al. 2020).

Already a single parental pathogen encounter can result in offspring protection from infectious disease (Shan et al. 2019) in a matching parent–offspring environment scenario. A mismatch in the microbial assemblage between paternal and offspring environments, though, might render TGIP a liability (Schmid‐Hempel 2021; Sheldon and Verhulst 1996): costs especially associated with constitutively TGIP‐induced immune vigilance will reduce offspring performance if the pathogen is not present anymore. However, even if the pathogen is still present in the offspring generation and TGIP‐induced immune vigilance is not constitutive but triggered only by a secondary encounter with that pathogen (a scenario where TGIP benefits should outweigh its costs), TGIP might become ineffective due to resource allocation trade‐offs: when additional environmental factors require organisms to mount a metabolically costly response to avoid severely detrimental effects, resources for a costly TGIP‐induced response to pathogens may not suffice (Goehlich et al. 2021; Metcalf, Roth, and Graham 2020; Roth and Landis 2017). Disentangling constitutive from inducible influences of parental immunological priming on offspring immune responses can thus illuminate how animals cope in a world of global change where emerging diseases can be a result of rapidly fluctuating abiotic conditions.

The immune system is in close reciprocal interaction with its natural microbiome. The microbiome can strengthen host immune responses, while the immune system shapes microbial niche colonization and composition (Leshem, Liwinski, and Elinav 2020; Litvak and Bäumler 2019). In early life, the initial microbial colonization of the gut is crucial for offspring performance and fitness, giving the neonate immune system assemblage and maturation a particularly important role (Barron and Young 2022; Fallet et al. 2022). The establishment of the gut microbiome can thus be expected to interact closely with TGIP, as the latter may potentially directly shape the vertical transfer of microbes supporting gut microbial colonization (Beemelmanns et al. 2019; Tanger et al. 2024). Closing the current knowledge gap of how immune priming shapes the microbiome and, *vice versa*, how the microbiome influences TGIP may provide insights into the heterogeneity of TGIP within and across populations (Prigot‐Maurice, Beltran‐Bech, and Braquart‐Varnier 2022).

Pipefishes inhabiting coastal seagrass ecosystems are exposed to a high diversity of microbes (Reusch et al. 2021), with *Vibrio* bacteria belonging to the most abundant bacteria both in the water column as well as in the pipefishes' gut microbiomes (Chibani et al. 2023; Crump and Bowen 2024; Möller et al. 2021; Roth, Keller, et al. 2012; Wendling et al. 2017). Over the season, *Vibrio* bacteria may change from mutualistic members of the microbiome to virulent disease agents through rapid changes in abiotic factors (e.g., temperature and salinity), reinforced by the increasing frequency of heatwaves in recent years (Claar and Wood 2020; Green et al. 2019; Roux et al. 2015). The pipefishes' unique male pregnancy makes them prime model systems for the investigation of transgenerational effects (Beemelmanns and Roth 2016a, 2016b, 2017; Roth, Klein, et al. 2012; Roth and Landis 2017; Schneider et al. 2022). While mothers may transfer their experience directly through the eggs, fathers prime their offspring through their intimate contact during their male pregnancy that evolved with a placenta‐like structure in *Syngnathus* and *Hippocampus*, among others (Beemelmanns and Roth 2016a, 2016b, 2017; Harada et al. 2022). Pipefishes and seahorses thus provide the unique opportunity to disentangle the effects of TGIP via egg production and pregnancy, which are usually intermingled within the female, by experimentally manipulating the maternal (via the egg) vs. the paternal (during pregnancy) immunological provisioning (Beemelmanns and Roth 2017; Roth, Klein, et al. 2012; Roth et al. 2018).

**FIGURE 1:**
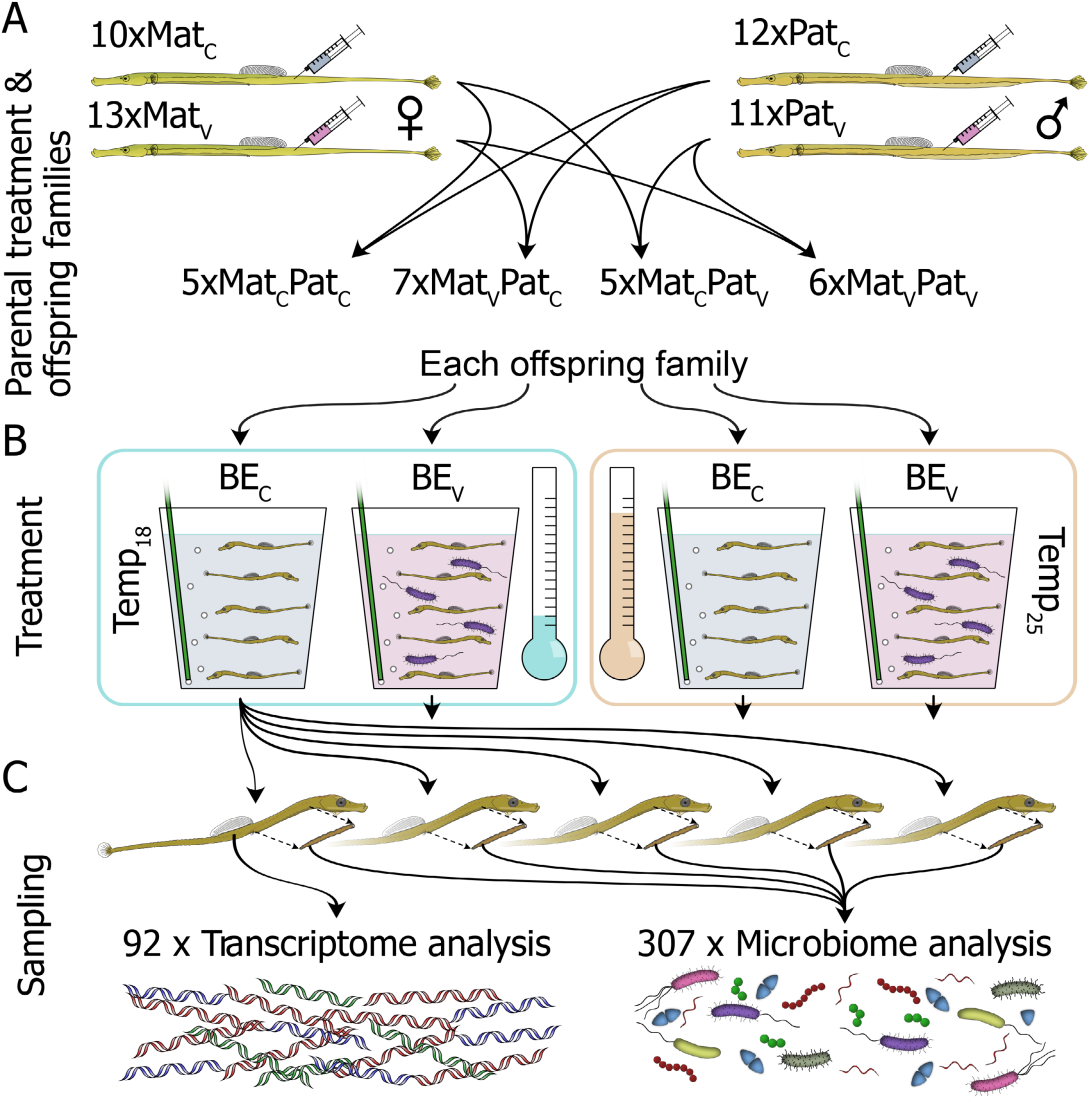
Experimental design for transgenerational immune priming. (A) Male and female pipefish were either vaccinated with heat‐killed *Vibrio* bacteria (Mat_V_ and Pat_V_) or sham‐treated with PBS (Mat_C_ and Pat_C_). Parents were mated and 23 juvenile sibling families (with ≥ 20 F1 each) were obtained, of which either none, one, or both parents had been vaccinated. (B) Per family, five juveniles were then kept in one of four treatments for 1 day: Seawater with or without *Vibrio*, at 18°C or 25°C. (C) Then, individuals were killed and their guts dissected. From each family treatment group, one juvenile without gut was used for RNA extraction and transcriptomics, while the guts of this individual as well as those of other individuals of the same group were used for DNA extraction and microbiome analysis. The given sample numbers are those finally considered throughout analyses.

In this study (Figure 1), we investigated TGIP in the broad‐nosed pipefish (*Syngnathus typhle*), aiming to unravel avenues of parental TGIP and its ecological relevance by testing five hypotheses: (1) TGIP (here, for the experimental *Vibrio* strain) has a protective effect for the offspring in microbially matching parent–offspring environments (here, the presence of the experimental *Vibrio* strain). (2) TGIP leads to differential gene expression even when no secondary exposure with the immune stimulus (no exposure to the experimental *Vibrio* strain—a non‐matching environment) occurs, reflecting a “cost” of TGIP. (3) TGIP enhances offspring protection specifically against the bacterial strain experienced by the parental generation, while immunological reactions against other strains/species remain unaffected. (4) Maternal and paternal TGIP lead to different response patterns in offspring upon bacterial exposure. (5) Strong abiotic stress (here, heat stress) experienced by offspring can compromise the effectiveness of TGIP.

Our data suggest that TGIP improved offspring defenses in matching biotic environments with the parental TGIP effects being distinct and not additive, and costs in mismatching *Vibrio* environments appear negligible. The heat stress scenario revealed how vulnerable this mechanism is, as it rendered parental effects virtually absent, probably as a result of a resource allocation trade‐off with a general heat stress response. This emphasizes the threat climate change‐induced heat waves pose to mechanisms natural populations employ to cope with swiftly changing microbial assemblages in their environment.

## 2 Material and Methods

### 2.1 Experimental Design and Fish Sampling

*Syngnathus typhle* individuals were caught in the seagrass meadow in Falckenstein (54.394497, 10.188988) in April 2020 (*prior* mating season), brought to the Helmholtz Centre for Ocean Research Kiel (GEOMAR) and kept in sex‐specific 200 L barrels with Baltic Sea water flow through. The temperature was gradually increased from 10°C to 18°C, and a day:night rhythm of 16 h:8 h was achieved over 3 weeks to approximate summer conditions in the Baltic Sea, inducing fish's mating readiness. Animals were transferred to 80 L aquaria attached to a common water circulation system and kept in same‐sex groups (Figure 1A). To induce immune priming in offspring, first, half of the males and females were vaccinated subcutaneously with 50 μL heat‐killed bacterial cells [10^8^ *Vibrio aestuarianus* bacteria/ml strain “I11Ma2”; originally isolated from a pipefish foregut caught in the Mediterranean Sea, Italy (Roth et al. 2011)] twice, with 1 week in between injections. One day after the second vaccination, eight mating pairs (“families”) were established for each of the intended parental mating groups in separate tanks attached to the same water flow through system (32 pairs in total): neither female (maternal treatment; “Mat”) nor male vaccinated (paternal treatment; “Pat”; group Mat_C_Pat_C_), only female vaccinated (Mat_V_Pat_C_), only male vaccinated (Mat_C_Pat_V_), and both female and male vaccinated (Mat_V_Pat_V_). The pairs were given 2 days for mating, after which females were removed, resulting in 23 families with sufficient offspring numbers after male pregnancy for the following experiments (final family numbers were Mat_C_Pat_C_: 5, Mat_V_Pat_C_: 7, Mat_C_Pat_V_: 5, Mat_V_Pat_V_: 6). Body length and weight were recorded for all parents.

Immediately after parturition, the offspring were transferred to a 1 L tank per parental family in a flow through system at 18°C, with the same day and night cycle as before and fed twice a day with live *Artemia salina* nauplii (Figure 1A). 9 to 10 days after birth, 20 offspring individuals per family were randomly selected for further treatments and, as offspring of different families varied in age (matings and parturitions were not perfectly synchronized across mating pairs), subsequent experiments were performed on three consecutive days to match offspring age. As family was treated as a random factor in our statistical analyses, a potential effect of the sampling day is also accounted for as all individuals per family were processed on the same day. The 20 individuals per family were split into four groups of equal sizes, and each group was moved into a beaker with 300 mL Baltic seawater, which was aerated by a weak air bubbler (Figure 1B). Two beakers per family were kept at 18°C (control temperature; “Temp_18_”), while the other two were kept at 25°C (heat stress situation; “Temp_25_”; temperature treatment = “Temp”). Finally, juveniles were subjected to a bacterial exposure (“BE”): 50 μL of a solution containing live *V. aestuarianus* (again strain I11M2; 10^8^ *Vibrio* bacteria/mL) was added to 50 mL rinsed 1 day old *Artemia* nauplii in suspension. After an incubation period of 30 min, 1 mL of this mixture was added always to one of the two beakers per family and temperature (“BE_V_”), while the other received the same amount of non‐enriched *Artemia* nauplii (“BE_C_”). After 1 day in the beakers, all juveniles were killed via a lethal dosage of MS222 (0.04% in PBS), and an overview photo was taken of all five juveniles per beaker to estimate treatment batch average body lengths using the segmented line tool in ImageJ (v.1.52k). Subsequently, juveniles were moved to RNAlater, kept for 1 day at 4°C, and then stored at −20°C. Juveniles were not monitored for a longer period of time as the transcriptomic response of key immune genes to an infection is most pronounced 6–48 h after an infection/immune stimulation in these fish (unpublished data). Additionally, the “natural” mortality (i.e., under the most ideal lab conditions that could be provided) and heterogeneity in growth rates of *S. typhle* juveniles massively increases after approximately 3 weeks of age, which is when a food transition occurs in captivity. This high mortality and growth rate heterogeneity would have confounded any estimate of survivorship in juveniles and would have substantially increased the required number of experimental animals. Unfortunately, keeping and treating five juveniles within a single beaker induced a nesting factor we cannot correct for (affecting the microbiota data set exclusively; see below); however, individual‐specific beakers were not feasible considering the large sample size. We therefore continued the analysis as if this effect would not exist, but acknowledge that conclusions from the microbiota data set have to be drawn cautiously. All experiments were conducted in accordance with local ethics regulations (University of Kiel Antrag §7 V242‐35168/2018).

### 2.2 Gut Dissections

To investigate the gut microbiome of juveniles, a transversal cut through the neck of each RNAlater‐stored specimen was conducted, still leaving the ventral body half connected to the trunk. Using forceps, the head (and attached tissues) was then pulled from the trunk, which led the ventral body wall to rip off directly posterior to the head, while the intestines remained connected to the head and instead got dislodged from the trunk close to the vent. Subsequently, the majority of the intestine was dissected from the head part, leaving only the most anterior part of the gut and neighboring organs, such as the liver, with the head. Intestines were stored in 100% EtOH for microbiome analyses, while all remaining tissues were immediately used for RNA extraction.

### 2.3 RNA Extraction, Library Synthesis, and Sequencing

Total RNA was extracted from one individual per family and treatment batch (i.e., “beaker”), leading to a total of 92 samples (23 families, with one individual for each of the two Temp and BE groups). For this purpose, remaining juvenile tissues (i.e., everything but intestines, which still includes immunogenic tissues, such as the head kidney, which are expected to respond strongly to an immune challenge) were taken immediately after gut dissection to homogenization in lysis buffer and processed using the Qiagen DNA/RNA All‐Prep Blood and Tissue Mini kit (Cat. No. 80284). Extracted RNA had an average concentration of 41 ng/μL and an average RIN of 9.7. mRNA library synthesis and sequencing (DNBseq, 150 bp PE, stranded) was conducted by BGI (HongKong, China), and on average, 58mio reads/sample were obtained, with all samples having > 30 mio reads, although some duplication levels were determined to be rather high, which might be reflected in some data noise.

### 2.4 Transcriptome Assembly

The RNA reads were quality‐trimmed with Fastp (v0.20.1; Chen et al. 2018). Alignments to the annotated genome were carried out with STAR (v2.7.9a; Dobin et al. 2013) in a two‐pass mode with the following options “‐‐outFilterIntronMotifs RemoveNoncanonical ‐‐outSAMunmapped None ‐‐outFilterMultimapNmax 1.” The count information per sample was obtained using TPMCalculator with options “‐c 150 ‐p ‐q 255 ‐e ‐a” and then merged into a single multi‐sample file with a script provided by the developer (tpmcalculator2matrixes.py). The output files from TPMcalculator were processed using a custom python script that for each *S. typhle* gene added an orthologous gene ID and gene description from *Danio rerio*, *Hippocampus erectus*, *Syngnathus acus*, and *Homo sapiens*. Missing orthology information was marked as NA. The orthology assignment was performed with Orthofinder (v2.4.0; Emms and Kelly 2019) using protein sequences from the following species: *Latimeria chalumnae*, *D. rerio*, *Xenopus tropicalis*, *Hippocampus comes*, *H. erectus*, *S. acus*, *S. typhle* (this annotation), *H. sapiens*, and *Mus musculus*. 26,072 genes were processed, and orthology was identified for *H. sapiens* in 14,435 genes, for *D. rerio* in 16,383 genes, for *S. acus* in 18,197 genes, and for *H. erectus* in 19,055.

### 2.5 (Pre‐)analysis of the Gene Expression Data Set and Removal of Dissection‐Related Variation

**FIGURE 2:**
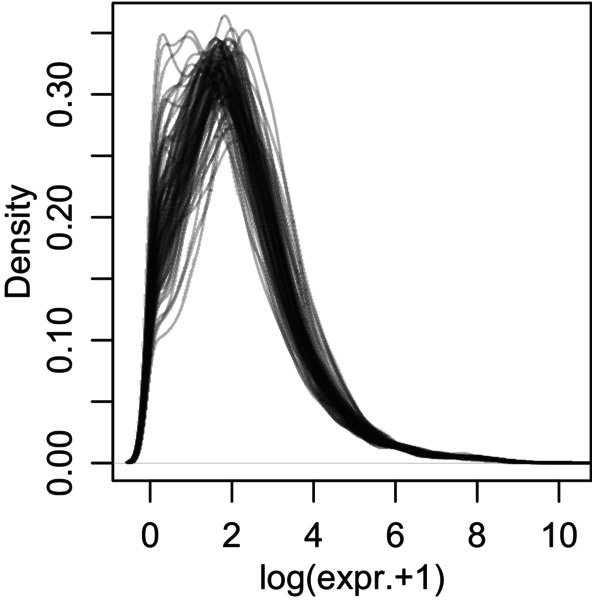
Density plots of all samples' raw TPM values. Each line represents one sample, and heterogeneous sample profiles suggest non‐homologous tissue compositions across samples.

A TPM (transcripts per million) expression table of 92 juvenile samples was imported into R (v. 4.1.3; R Core Team 2021) for downstream analyses, and genes that were expressed in fewer than three samples were discarded, leaving 19,055 genes. Density plots of gene expression profiles were generated for each individual (Figure 2), revealing rather heterogeneous expression patterns, which may be indicative of non‐homologous tissue sampling.

**FIGURE 3:**
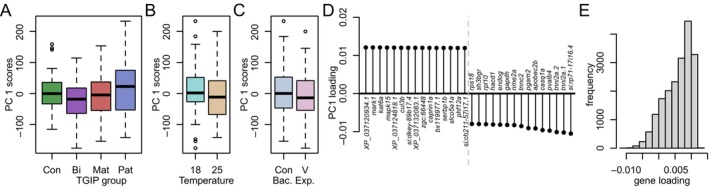
PCA plots of uncorrected TPM data. (A–C) PC1 scores did not show differentiation for any intended treatment. (D) Gene loadings on PC1 suggested that genes involved in muscle development and metabolism are highly loaded on PC1. (E) A histogram of gene loadings on PC1 showed a strongly skewed distribution.

To explore main axes of variation in the gene expression data set, a principal component analysis (PCA) was used on the log(gene_expr+1)‐transformed, centered, and scaled gene expression data set (Figure 3). When investigating the scores and loadings of PC1, we noticed that none of the major treatments seemed to be reflected on PC1 scores (Figure 3A–C), using Dunnett's Test with the non‐primed group as control as the reference level to test for differences among parental treatments, and t‐tests to test for differences between Temp and BE levels (all *p* > 0.1; package “DescTools,” v. 0.99.47; Signorell et al. 2023). Additionally, genes heavily negatively loaded on this PC unexpectedly appeared to be associated with energy metabolism (and, to a lesser degree, muscle tissue; Figure 3D), and a histogram of the loadings suggested an atypical negative skew, indicating that PC1 might reflect unintended variation present in the data set (Figure 3E).

**FIGURE 4:**
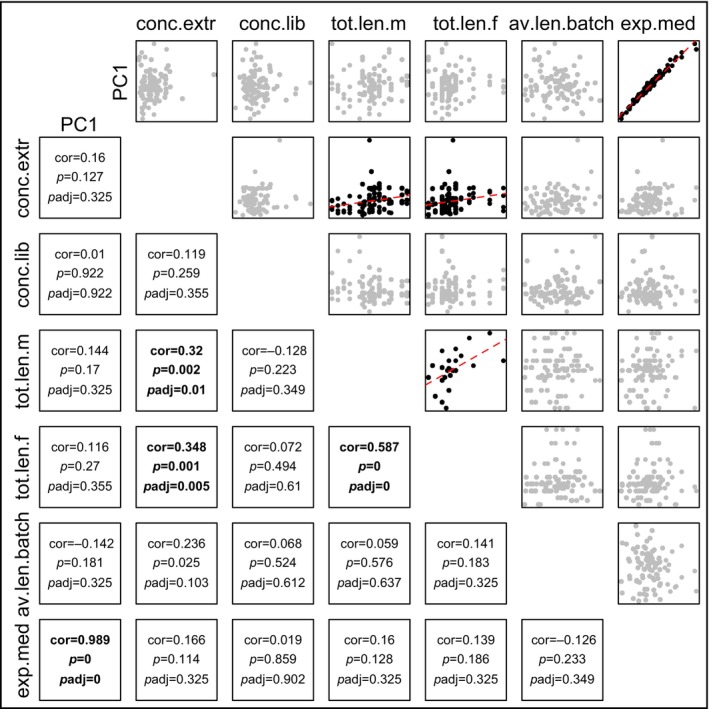
Correlations between the uncorrected PC1 and possible confounding variables. Scatterplots with regression lines and Spearman correlation statistics, incl. fdr‐corrected *p*‐values, for considered confounding variables with each other and the uncorrected PC1. Considered variables were: RNA concentration after extraction (“conc.extr”), RNA concentration as measured directly before library synthesis (“conc.lib”), father body length (“tot.len.m”), mother body length(“tot.len.f”), average body length of offspring batch (“av.len.batch”), and median of samples' expression distribution (“exp.med”; Figure 2).

A set of possible technical and biological covariates was investigated for correlations with PC1, which would suggest an influence on overall gene expression patterns (Figure 4). These included per sample (i) the total RNA concentration after extraction and (ii) after shipping (as determined by the sequencing company), (iii) the respective juvenile's father and (iv) mother total body length, (v) the average body length of juveniles of the respective batch (i.e., “beaker”), and (vi) the median of gene expression across all genes (which reflects the variation in gene expression profiles mentioned above, Figure 2). These variables and PC1 were correlated to each other using Spearman Rank‐sum correlations, and the obtained *p*‐values were corrected using the false discovery rate (FDR) method. The results revealed that PC1 scores were highly significantly associated with the median of the log gene expression (*ρ* = 0.99, *p*.adj < 0.001), indicating that the variation in gene expression profiles displayed in Figure 3 indeed is the conceptually unintended major source of variation in the data set. To understand the cause of this variation, PC1 loadings were inspected. The large majority of genes (16,979) showed positive loading, while much fewer (2076) showed negative loading (Figure 3E). 1% of most positively loaded genes (*n* = 191) were analyzed for Gene Ontology (GO) biological process enrichment. The human GO annotation was used, as it is the most comprehensive, even though not for all genes, human ortholog annotations were present (*n* = 165 with a human gene annotation; *n* = 157 of those with GO annotation), and their function in humans (and thus GO association) may be different compared to fish (corresponding results for GO analysis using *D. rerio* annotations are reported in Data files uploaded to Dryad, but remained less conclusive and thus is not discussed here). We tested for GO enrichment using the gost(…, ordered_query = F, organism = “hsapiens,” significant = T, correction_method = “gSCS,” sources = c(“GO:BP”), custom_bg = all_expressed_genes, domain_scope = “custom”) function (“gprofiler2” v.0.2.1; Kolberg et al. 2020; all_expressed_genes include 14,435 human orthologs). Eight terms were found significantly enriched, of which four were cellular regulation/organization‐related, while the remaining were generic regulatory processes, such as chromatin organization. When this was done with the 1% most negatively loaded genes (*n* = 132 of which had a human ortholog and *n* = 121 of these a GO annotation), 21 processes were identified, and almost all appeared to be related to energy metabolism. These GO terms, the overall skew in gene loading frequencies toward positive loadings (Figure 3E), and the common energy metabolism genes with negative loading (Figure 3D; also, several muscle‐related GO terms narrowly missed significance) suggest that variation reflected in PC1 is likely linked to differences in tissue composition (possibly reflecting a varying amount of the functionally distinct foregut portion of the gut) of samples. This is likely also reflected in the heterogeneous expression profiles (Figure 2), possibly as a result of inconsistencies during gut dissections.

To correct for this effect and to identify whether and how the intended treatment factors affected gene expression, an unconventional analysis approach was chosen over more orthodox pipelines. The aim was to remove unintended variation in gene expression reflected in this PC1 in our subsequent statistical analysis. The applied pipeline thus proceeded as follows: individual genes were investigated, and a gene was removed from the pipeline if it was not expressed in at least all but one individual of at least two treatment groups (from among the 16 treatment combinations), and mean log(gene expression+1) levels among individuals expressing the gene had to be at least 0.1 to be deemed meaningful. In this manner, 1567, 7, and 832 genes were excluded for being too low expressed, being expressed in too few samples, or both, respectively, leaving 16,649 to be considered in the following steps. Then, for each gene, a linear model was computed using the aforementioned log‐transformed gene expression as the dependent variable and PC1 scores as the independent variable, and its residuals were stored.

### 2.6 Pairwise Comparisons of Gene Expression

Gene residuals were used as a dependent variable to compute gene‐wise linear mixed models that also included treatment variables and their interactions as independent terms (model in R: lme(log_gene_expression_residuals ~ Mat × Pat × BE × Temp, random = ~1|family, method = “REML”); package “nlme,” v. 3.1‐161 Pinheiro et al. 2013; Pinheiro and Bates 2006). Due to their large number, individual models were not inspected for violations of model assumptions. For subsequent pairwise comparisons, the function glht() (package “multcomp,” v.1.4‐19; Hothorn et al. 2016) was used to test seven treatment groups (Mat_V_Pat_C_BE_C_Temp_18_, Mat_C_Pat_V_BE_C_Temp_18_, Mat_C_Pat_C_BE_V_Temp_18_, Mat_C_Pat_C_BE_C_Temp_25_, Mat_V_Pat_C_BE_V_Temp_18_, Mat_C_Pat_V_BE_V_Temp_18_, and Mat_V_Pat_V_BE_V_Temp_18_) against a reference group (Mat_C_Pat_C_BE_C_Temp_18_) based on the mixed model. Estimates and raw *p*‐values were computed for each pairwise comparison. Also, to compute the corresponding comparisons with the reference temperature of 25°C, the aforementioned procedure was repeated with the reference level of the Temp factor being set to 25°C. Raw *p*‐values were corrected for multiple testing using the p.adjust() function and the “fdr” method per comparison across all genes. For each comparison that yielded genes with fdr‐corrected *p*‐values below *α* = 0.05, Gene Ontology (GO) terms were assigned to all genes when available and tested for enrichment as described before.

### 2.7 Average Immune Cell Expression Rank Analyses

To explore if sets of significantly expressed genes were associated with immune functions, we investigated their association with immune cells. First, a reference single cell expression data set based on human data was downloaded from the human protein atlas website using the R package “HPAanalyze” (v. 1.16.0; Tran et al. 2019). The “RNA single cell type” data set was chosen, which contains normalized expression data for 79 different annotated cell types (incl. “undifferentiated”) collated from various studies' data sets. Of these cells, 13 were identified as being immune cell‐related, and while “dendritic cells” were not assigned to one of the two branches of the immune system, those specific to the innate branch were “granulocytes,” “Hofbauer cells,” “Kupffer cells,” “Langerhans cells,” “macrophages,” “microglial cells,” and “monocytes,” while those specific to the adaptive branch were “B‐cells,” “NK‐cells” (natural killer cells), “Plasma cells,” “T‐cells,” and “thymic epithelial cells.” All 14,435 human orthologs from our study were matched by Ensembl ID to the genes within the downloaded database. For each of these genes, we aimed to estimate its association in expression (in humans) to immune cell types, i.e., a high association reflects that a gene is predominantly expressed across immune cell types and rarely or only at low levels in other cell types. For this, first, cell types within the downloaded data set with no gene expression associated with them were excluded and the remaining cells were sorted according to their average normalized expression values (in human) and ranks were assigned (the cell type with the highest expression value obtained the highest rank). Obtained rank numbers were then divided by the number of non‐zero cells, and the rank sum values of the designated immune cells (IM value), those assigned to the innate (IN value), and adaptive immune system (AD value) were calculated, resulting in three values that reflect how strong a given gene's expression is associated with cells of the respective type relative to all others (higher values indicate stronger association). Additionally, to illustrate if a gene is associated rather with cell types of one branch of the immune system over the other, the rank sums of the adaptive cell types were subtracted from the rank sum of the innate cell types, resulting in a value (IA) in which more positive numbers indicate higher gene expression association with cell types of the innate immune system, while more negative values indicate this for cell types of the adaptive immune system. While these values by themselves are not very informative, their distribution across all genes of the background gene set served as a reference to investigate the distribution of these values in sets of target genes, e.g., sets of differentially expressed genes (DEGs). To determine if target gene sets contained genes that on average had different IM, IN, AD, or IA values compared to the reference background, Wilcoxon signed rank tests were performed and the distribution of ranks was plotted for both backgrounds and target gene sets for IM and IA values. Still, for these analyses, it should be kept in mind that gene sets are not independent of each other, many genes overlap among them, and *p*‐values derived from Wilcoxon signed rank tests were not corrected. Also, not all genes had a human annotation and thus actual sample sizes for comparisons were somewhat smaller than what the DEG lists suggest.

### 2.8 Microbiome Sequencing, Assembly, and Analysis

317 gut samples were processed using the Qiagen DNA Blood and Tissue kit (Cat. No.: 69504). Obtained DNA was sent to the IKMB sequencing center, Kiel, Germany, which performed library preparation and Illumina MiSeq amplicon paired‐end sequencing of the V3‐V4 bacterial 16S rRNA region randomly distributed to three runs (run 54: *n* = 30, 55: *n* = 8, and 57: *n* = 269). Sequences were demultiplexed, conjoined with their respective metadata and processed, quality‐filtered, and analyzed (package “QIIME2,” v.2021.8; Bolyen et al. 2019). Correction of the fastq files was achieved using the DADA2 software within the QIIME2 pipeline, which included denoising, merging, chimera slayer fitting, and trimming of the sequences (“ASVs”). Due to declining quality scores, sequences were truncated 50 bases from forward and 70 bases (run 54) or 100 bases (run 55, run 57), respectively, from reverse reads. Taxonomy was assigned using a Naive Bayes classifier trained on a SILVA138 99% OTU full‐length sequence database. Microbiota count and metadata of all 317 samples were imported into R using the function qza_to_phyloseq() (package “qiime2R” v.0.99.6; Bisanz 2018; 89 samples of these were also used for gene expression analysis), with reads being assigned to 7100 microbial taxa (ASVs). Samples with log(counts+1) ≤ 8 (~3000 reads) were excluded after histogram inspection (leaving 307 samples; minimum sample size per subgroup was *n* = 13 and maximum number *n* = 23). The remaining samples' counts were normalized by dividing them by the respective sample's mean count across taxa and then multiplying them by the mean count across all samples and taxa. Thus, when the text refers to “counts” for brevity, it means “normalized counts” or “relative abundance.”

**FIGURE 5:**
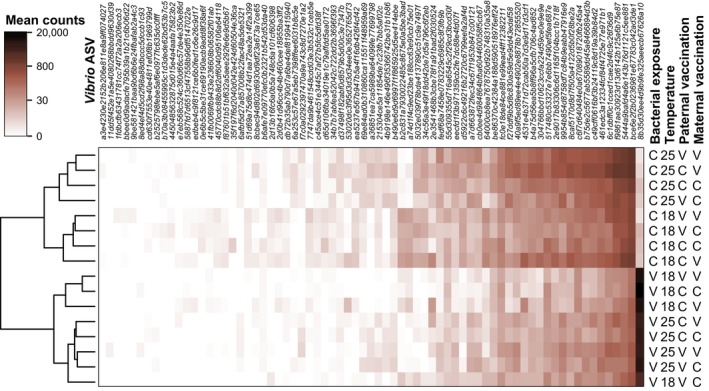
Heatmap of *Vibrio* strain counts. Strains have been sorted according to their average count inclining from left to right and only those with on average > 1 count/sample are shown. The strain most to the right has the highest average count and also a marked difference between samples treated with the experimental *Vibrio* strain and the control group, indicating that it is the experimental *Vibrio* strain.

The mean log(count+1) of all *Vibrio* ASVs were visualized using a heatmap (Figure 5). One strain (formerly two ASVs of identical barcode sequences were merged into one) not only showed the highest overall abundance among ASVs (16.5% of all microbiome counts) but also featured a marked difference in abundance between bacterial exposure groups, indicating that it represents the experimental strain. The experimental strains abundance across samples that also had RNA‐seq data (*n* = 87) did not correlate with the bias‐indicating original RNA‐seq PCA PC1, suggesting that no correction was required (*S* = 127,812, *p*‐value = 0.1273). Treatment effects on the experimental *Vibrio* counts were analyzed by first log‐transforming all 307 samples (log(counts+1)), followed by visual inspection across treatment groups; nine outliers were excluded. The remaining samples' transformed *Vibrio* counts were used as the dependent variable in a linear mixed model (lme() function in R, with the maximum likelihood method). A qq‐plot on residuals was used to investigate normality of residuals, and some deviation from normally distributed residuals was noted, mostly due to eight outliers. Attempts to remove these outliers led to model results very similar to those including outliers (see below), except for stronger and more significant effects, illustrating their limited effect on the conclusions drawn from the model. Outliers were therefore retained. As in the gene expression analyses, independent variables were the factors Mat, Pat, BE, and Temp, and all their interactions. A random intercept for the family factor was included (“random = list(~1|family)”), but also a variance structure for the BE treatment (“weights = varIdent(form = ~1|BE)”), compensating for the heterogeneous variance due to this treatment. As the focus of the analysis was to determine if parental priming induced by parental vaccinations affects microbe counts, the *Vibrio* exposure treatment level (and not the control) was chosen as a reference level for the BE factor. The Anova() function was used again to obtain an ANOVA table, and type III ANOVA was chosen, as interactions rather than main effects were of central interest.

Adding large amounts of the experimental *Vibrio* may affect the obtained microbiome sequencing signal of the respective treatment group (BE_V_; i.e., if a large proportion of read depth was claimed by the experimental *Vibrio*, relatively fewer reads might have represented remaining *Vibrio* and *non‐Vibrio* strains). Using Wilcoxon tests, we tested whether BE_V_ samples showed a higher read count than BE_C_ samples when all *Vibrio* strains were summed up per sample. Then, the same was tested after subtracting the experimental *Vibrio* counts in both groups. Subsequently, the sum of counts from samples that were exposed to the experimental bacteria (BE_V_) was divided by the sum of all samples (BE_C_ + BE_V_) to estimate *Vibrio* treatment count bias in a proportion (the result indicated which proportion of reads came from BE_V_ samples). To understand if the obtained proportion was similar across microbiota genera (suggesting sequencing depth was a limiting factor across samples), for each sample, the sum of all counts of strains belonging to each genus identified in this study was calculated (853 genera). Then, for each genus, the proportion of counts found in samples exposed to the experimental *Vibrio* was determined. We excluded genera that had counts in less than 5% of the 307 individuals considered, and we excluded the genus *Vibrio*, leading to 226 genera considered. Finally, a one‐sample Wilcoxon test was used to test if the genus *Vibrio* was showing a significantly higher treatment bias than other genera.

For further analyses of the remaining microbiome, first, counts of the experimental *Vibrio* strain were removed and samples re‐standardized. Then, all counts from within each microbial family taxon were summed up per individual and families with a total of less than 10 counts were removed (332 microbial families remaining). To test if the immune challenge phenotypes elicited by parental immune priming also affected this remaining microbiome, two ANOSIM analyses were conducted using Bray–Curtis dissimilarity matrices: one on microbiome abundances from samples without bacterial challenge at 18°C (Mat_C/V_Pat_C/V_BE_C_Temp_18_), and one on samples with this exposure (Mat_C/V_Pat_C/V_BE_V_Temp_18_; anosim(); “vegan” package v.2.6‐2), for both of which the factor level combinations of Mat and Pat were treated as one factor with four levels. The Shannon diversity index was calculated per individual using log(counts+1), and its correlation with experimental *Vibrio* counts was assessed using a Spearman rank correlation. Additionally, to estimate how treatment groups affected alpha diversity, two linear mixed models were computed using the alpha diversity as the response variable and Mat, Pat, and Temp as predictor variables, plus either BE or the experimental *Vibrio* log(counts+1), as well as all interactions (“family” was added as a random intercept). For both models, model selection using the stepAIC() function was conducted, and while both models yielded similar BIC values (200.97 and 199.49, respectively), *Vibrio* counts were suggested as a slightly better predictor than BE, and it was therefore evaluated using the Anova(…, type=“III”) function.

## 3 Results

### 3.1 Transcriptome Analyses

**FIGURE 6:**
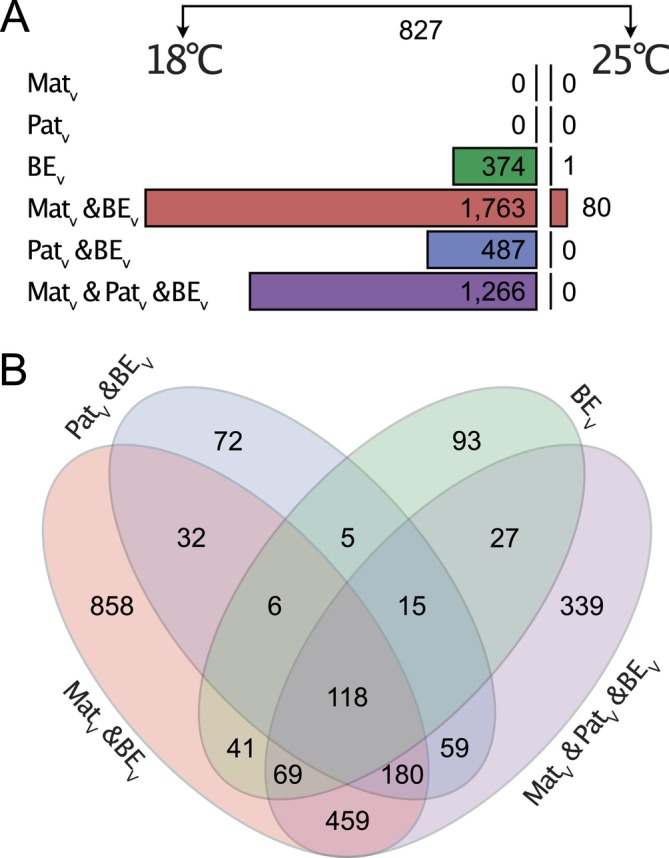
Numbers of differentially expressed genes of pairwise comparisons based on gene expression mixed models. (A) Numbers of significantly differentially expressed genes (after fdr correction) between focal groups and the Mat_C_Pat_C_BE_C_Temp_18_ (left side) or Mat_C_Pat_C_BE_C_Temp_25_ (right side) reference group. (B) VENN diagram of shared differentially expressed genes among comparisons for comparisons with the 18°C reference group.

Gene expression differences between selected treatment groups and a reference group were evaluated for each temperature treatment separately using pairwise comparisons based on gene‐wise linear mixed models (Figure 6). Using the group Mat_C_Pat_C_BE_C_Temp_18_ as a reference, neither the maternal nor paternal vaccination by itself induced significant changes in offspring gene expression at 18°C after fdr correction when offspring were not exposed to the experimental bacterium (Figure 6A). In offspring from non‐vaccinated parents, bacterial exposure (Mat_C_Pat_C_BE_V_Temp_18_) led to 374 DEGs at 18°C. In contrast, when offspring of a vaccinated mother was exposed to the experimental bacteria, 1763 DEGs were identified (Mat_V_Pat_C_BE_V_Temp_18_), while 487 DEGs were found if only the father was vaccinated (Mat_C_Pat_V_BE_V_Temp_18_), and 1266 genes differed in expression compared to the reference when both parents were vaccinated at 18°C (Mat_V_Pat_V_BE_V_Temp_18_). When the same comparisons were conducted with offspring treated at 25°C (including the reference: Mat_C_Pat_C_BE_C_Temp_25_), bacterial exposure of the offspring (Mat_C_Pat_C_BE_V_Temp_25_) led to only a single DEG. Furthermore, the offspring from vaccinated mothers (Mat_V_Pat_C_BE_V_Temp_25_) showed 80 DEGs after bacterial exposure at 25°C when compared to the reference—all other considered comparisons did not yield any DEGs. When gene expressions from non‐challenged offspring from unvaccinated parents were compared between temperature treatments, 827 DEGs were identified (Mat_C_Pat_C_BE_C_Temp_18_ vs. Mat_C_Pat_C_BE_C_Temp_25_). Only 27 of these were also among the previously mentioned 374 genes that responded to the bacterial exposure at 18°C (Mat_C_Pat_C_BE_C_Temp_18_ vs. Mat_C_Pat_C_BE_V_Temp_18_), although 26 of these 27 genes showed an expression change in the same direction relative to the reference group (Mat_C_Pat_C_BE_C_Temp_18_ in both cases).

**FIGURE 7:**
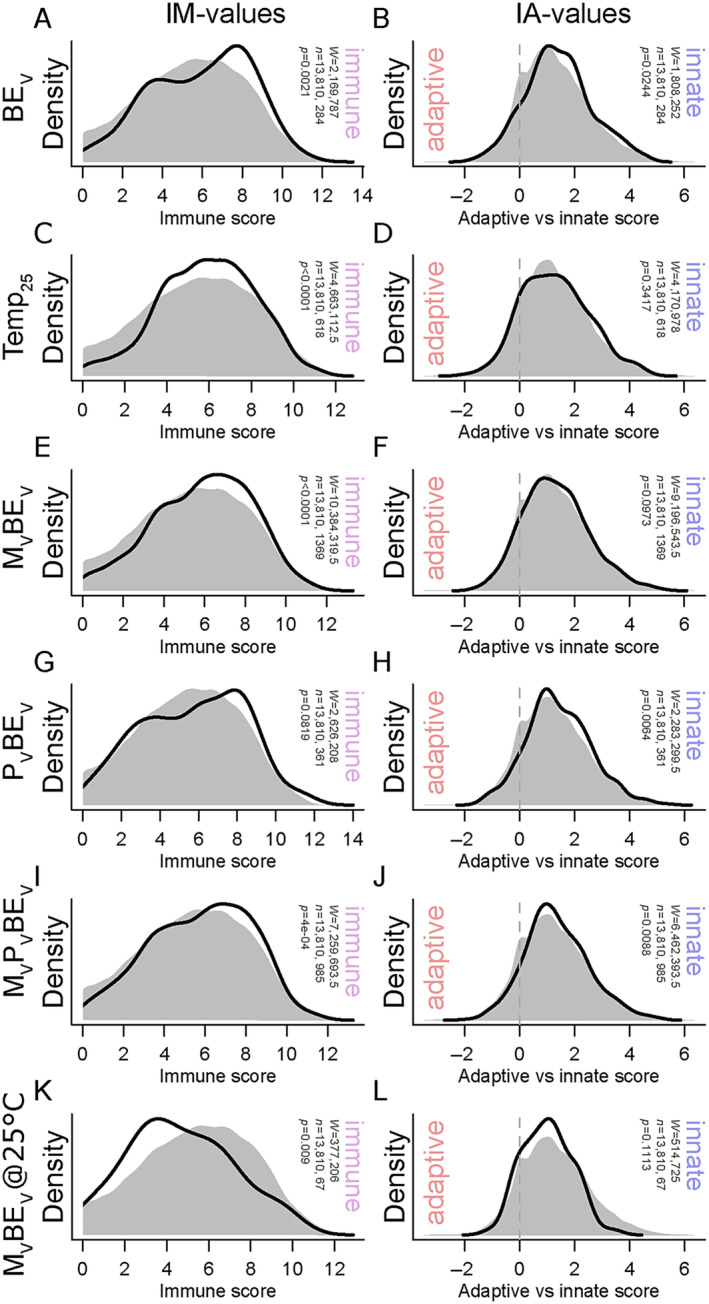
Average immune cell expression rank analyses of target gene sets. (A, C, E, G, I, K) Distribution of IM values (these reflect gene sets' associated with all immune cells) and (B, D, F, H, J, L) IA values [these reflect gene sets' association bias toward the innate (positive values) or adaptive immune branch (negative values)] of significantly differentially expressed genes of selected pairwise comparison shown as black lines (see Figure 6). Reference groups are (A–J) Mat_C_Pat_C_BE_C_Temp_18_ or (K, L) Mat_C_Pat_C_BE_C_Temp_25_. Gray shading represents the distribution of these values among all genes considered in the analysis with human annotation (13,810 genes). Significant differences in median values between sets of significant genes and the background (all genes) were determined using Wilcoxon tests.

To evaluate if sets of DEGs derived from pairwise comparisons were enriched in genes highly expressed in immune cells, we used a custom average immune cell rank analysis (see Section 2). For genes differentially expressed between juveniles from non‐vaccinated parents without bacterial exposure and juveniles from non‐vaccinated parents with bacterial exposure (Mat_C_Pat_C_BE_C_Temp_18_ vs. Mat_C_Pat_C_BE_V_Temp_18_: 374 genes; Figure 7A,B), a significant increase in IM values was detected (*p* = 0.002; suggesting an association with immune cells), as well as significantly higher AD (*p* = 0.032; suggesting an association with cells of the adaptive immune branch), IN (*p* = 0.002; suggesting an association with cells of the innate immune branch), and IA values (*p* = 0.024; suggesting a stronger association with cells of the innate than adaptive immune branch). For the 827 DEGs between the 18°C and the 25°C control groups (Mat_C_Pat_C_BE_C_Temp_18_ vs. Mat_C_Pat_C_BE_C_Temp_25_), we found, on average, significantly higher IM values compared to the background (*p* < 0.001; Figure 7C,D), which was also true for AD (*p* < 0.001) and IN (*p* < 0.001) values, but not IA (*p* > 0.05) values, suggesting that there is no bias toward one of the immune branches.

Similarly, genes differentially expressed between the bacterial exposure individuals without parental vaccination and those with maternal vaccination (Mat_C_Pat_C_BE_C_Temp_18_ vs. Mat_V_Pat_C_BE_V_Temp_18_: 1763 genes) also showed on average higher IM, AD, and IN scores than the background (all *p* < 0.001; Figure 7E,F), while there was a trend in AI values between this gene list and the background, suggesting that possibly this effect is stronger in the innate immune branch than the adaptive one (*p* = 0.097). In contrast, genes differentially expressed between the control group and paternally vaccinated and bacterial exposure individuals (Mat_C_Pat_C_BE_C_Temp_18_ vs. Mat_C_Pat_V_BE_V_Temp_18_: 487 genes) were significantly increased in IN and AI values compared to the background (*p* = 0.02 & *p* = 0.006, respectively; Figure 7H), suggesting that DEGs were indeed specifically linked to cells of the innate immune system. Wilcoxon signed rank tests suggested a difference in neither IM nor AD values relative to the background, but the slightly bimodal shapes of the DEGs' distributions (Figure 7G for IM) may differ in variance to the background rather than median—which cannot be detected using our testing regime.

GO enrichment was explored for genes with significant differential expression in pairwise comparisons (Figure 6A), and only for DEGs of one pairwise comparison enriched terms could be identified (with both human and zebrafish annotations; both annotations can be found in data files deposited on Dryad). With human annotation, the six terms enriched in the temperature comparison (i.e., non‐bacteria exposed offspring from non‐vaccinated parents 18°C vs. 25°C) are linked to catabolic and metabolic processes, but also blood regulation (e.g., “regulation of body fluid levels” and “blood coagulation”). Using zebrafish annotations, additional GO terms were enriched, suggesting immune system involvement, for example, “complement activation,” “complement activation, alternative pathway,” “killing of cells of another organism,” and “disruption of cell in another organism.”

Genes differentially expressed between juveniles without parental vaccination and no bacterial exposure, and those bacterial exposure individuals with biparentally vaccinated parents (Mat_C_Pat_C_BE_C_Temp_18_ vs. Mat_V_Pat_V_BE_V_Temp_18_: 1266 genes; Figure 7I,J) showed significantly increased IM, AD, IN, and AI values (all *p* < 0.05), suggesting that expression of DEGs was over‐proportionally associated with cells of both branches of the immune system, but also that this effect is significantly stronger for the innate branch compared to the adaptive branch. Finally, the genes that differed at 25°C between the control group and bacterial exposure offspring from vaccinated mothers (Mat_C_Pat_C_BE_C_Temp_25_ vs. Mat_V_Pat_C_BE_V_Temp_25_: 80 genes; Figure 7K,L) showed significantly smaller average IM and IN values (and a trend toward smaller AD values *p* = 0.052) compared to the background, suggesting that expression of these genes was less associated with immune cells than in the background. In summary, we identified an overall trend for DEGs at 18°C to be associated with genes of the immune system. Especially for the BE treatment and the paternal vaccination groups, this affects the two branches of the immune system differently (in both cases, the innate branch appears to be more associated to DEGs). In contrast, the few identified DEGs in the maternal vaccination group at 25°C were actually less associated with immune genes than the background.

### 3.2 Microbiome Analyses

**FIGURE 8:**
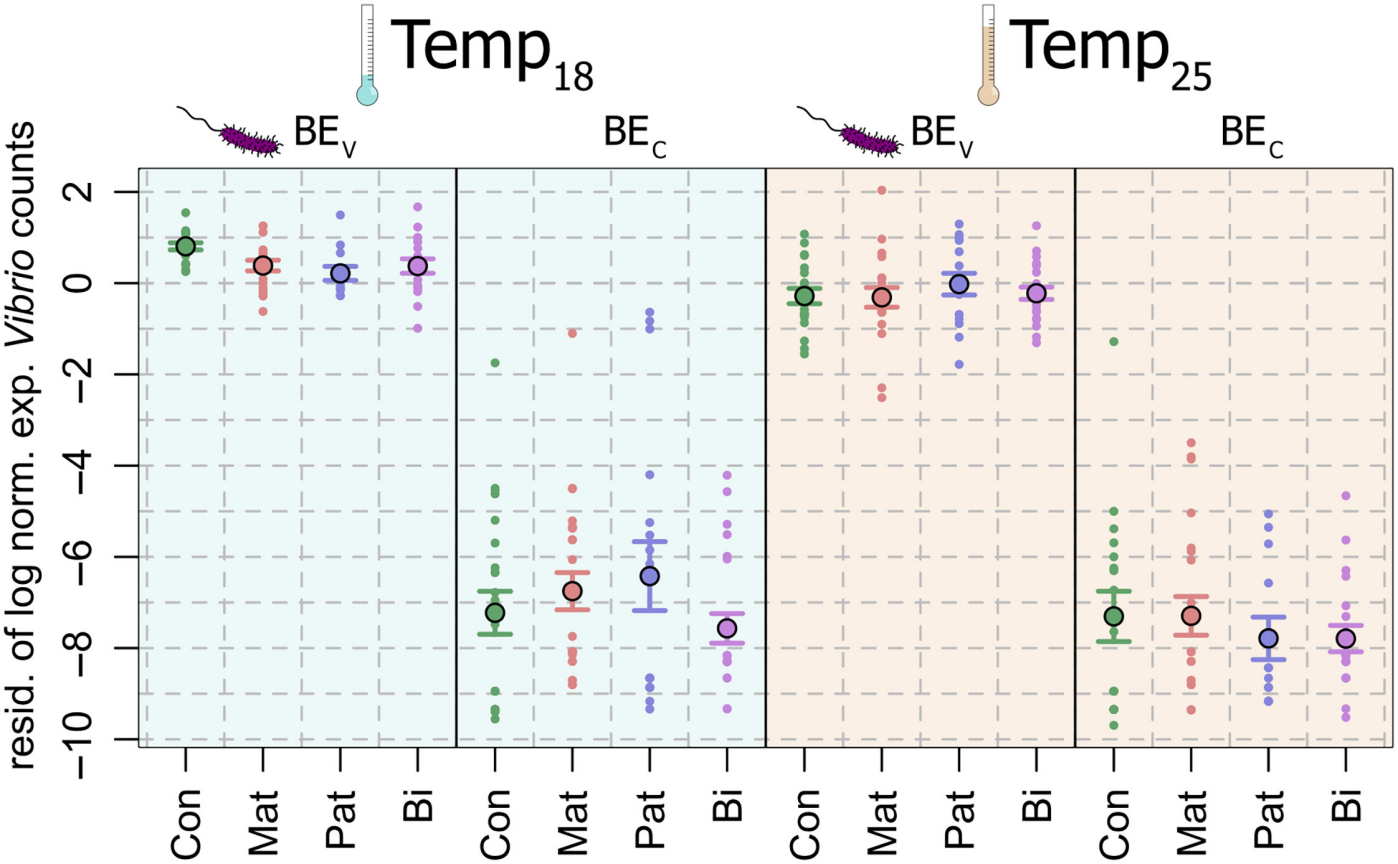
Relative experimental *Vibrio* abundances. Mean and standard error of family residuals of log‐transformed relative experimental *Vibrio* counts across experimental treatment groups.

Complementary to transcriptome analyses, the gut microbiome of 307 individuals was investigated and experimental *Vibrio* counts were first investigated in isolation. Normalized experimental *Vibrio* counts were used as a dependent variable in a mixed model that identified significant negative main effects for the factors BE, Temp, Mat, and Pat (all *p* < 0.05; Table 1; Figure 8). As expected, the most significant and largest effect among these was the BE effect, confirming that treated juveniles showed substantially higher relative abundance of the experimental *Vibrio* strain. A trend (*p* = 0.076) toward a positive interaction of Mat and Pat might suggest that parental vaccination effects are not additive but rather redundant. A significant interaction of the Pat and Temp factors (*p* = 0.034) with a positive estimate indicates that the negative effect of paternal vaccination on *Vibrio* abundance in the guts of offspring experimentally exposed to bacteria was not maintained at higher temperatures and possibly was even inverted. Surprisingly, the model also indicates a trend that offspring from vaccinated parents but with no bacterial exposure might have had higher numbers of the experimental *Vibrio* than offspring with non‐vaccinated parents: the interactions of Pat and BE and Mat and BE, while not reaching significance, show positive estimates and quite low associated *p*‐values (0.054 and 0.123, respectively; but Figure 8 illustrates that some outliers might have driven this pattern). In contrast, the offspring from biparentally vaccinated parents do not show this potential effect, as is reflected in a significant interaction of Mat, Pat, and BE (*p* = 0.022). The associated negative slope thus compensates (to some extent) for the aforementioned trends with positive estimates, that is, offspring from biparentally vaccinated parents that were not exposed to bacteria have the same low levels of the experimental *Vibrio* as offspring from non‐vaccinated parents. A significant interaction of Pat, BE, and Temp (*p* = 0.047) with a negative slope illustrates that at 25°C, non‐challenged paternally vaccinated individuals showed lower experimental *Vibrio* abundance than at 18°C, at which this group appeared to have a higher abundance compared to the BE_C_ control group. The paternal vaccination effect was thus more dependent on environmental temperature than the maternal effect.

**TABLE 1:** Statistical summary of the linear mixed effects model with experimental *Vibrio* log(count+1) as the dependent variable and Mat, Pat, BE, and Temp, as well as all interactions as independent variables. *p < 0.05; **p < 0.01; ***p < 0.001.

Variable	Estimate	*χ* ^2^	df	Pr(> *χ* ^2^)
(Intercept)	10.24	1376.71	1	< 0.0001***
Mat_V_	−1.14	9.13	1	0.003**
Pat_V_	−1.02	5.87	1	0.015*
BE_C_	−8.1	318.67	1	< 0.0001***
Temp_25_	−1.14	22.76	1	< 0.0001***
Mat_V_:Pat_V_	1	3.15	1	0.076
Mat_V_:BE_C_	0.97	2.38	1	0.123
Pat_V_:BE_C_	1.34	3.72	1	0.054
Mat_V_:Temp_25_	0.45	1.84	1	0.175
Pat_V_:Temp_25_	0.8	4.47	1	0.034*
BE_C_:Temp_25_	1.08	2.58	1	0.108
Mat_V_:Pat_V_:BE_C_	−2.15	5.28	1	0.022*
Mat_V_:Pat_V_:Temp_25_	−0.71	2.01	1	0.156
Mat_V_:BE_C_:Temp_25_	−0.94	1.02	1	0.312
Pat_V_:BE_C_:Temp_25_	−2.08	3.96	1	0.047*
Mat_V_:Pat_V_:BE_C_:Temp_25_	2.31	2.79	1	0.095

**FIGURE 9:**
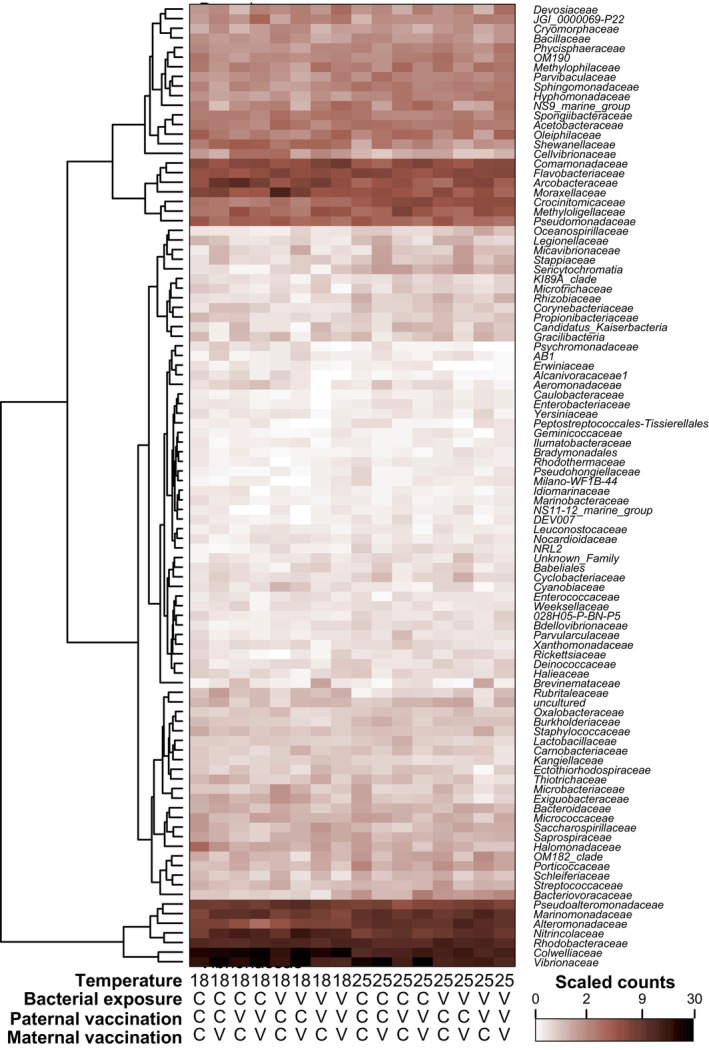
Heatmap of most abundant microbe families. Most abundant 100 microbe families across samples after count normalization and scaling. Color shades indicate sums per treatment group combination.

To determine if parental vaccinations affected gut microbiota compositions beyond the experimental *Vibrio* strain, all other strains' counts were summed up on the family taxon level per sample and mean count sums were calculated per factor level combination across samples (Figure 9). The highest relative abundance among families (excluding the experimental *Vibrio* strain) were (still) *Vibrionaceae* (28% of all counts), *Colwelliaceae* (23% of all counts), and *Rhodobacteraceae* (8% of all counts; all others < 6%). Based on this family‐level table (excl. experimental *Vibrio* counts), the Shannon diversity index was calculated per sample, which was found to be negatively correlated to the log abundance of the experimental *Vibrio* strain (Spearman correlation: *S* = 5,495,530, rho = −0.14, *n* = 307, *p* < 0.0144), an effect possibly (at least partly) caused by the reduced sequencing read numbers in bacteria‐exposed juvenile samples that were available for non‐experimental microbiota strains. A mixed model also confirmed that the Shannon diversity was negatively correlated with the variable “exp. *Vib*. log(counts+1)” (*p* = 0.0009), and the higher temperature group was associated with higher Shannon diversity values (Table 2). While the paternal vaccination effect was also retained in the final model, by itself it did not attain significance (*p* = 0.11); however, its interaction with the experimental *Vibrio* count variable (*p* = 0.0087) could suggest that a paternal vaccination effect can affect the Shannon diversity dependent on the prevalence of the experimental *Vibrio* strain: the more experimental *Vibrio*, the more positive the effect of paternal vaccination on alpha diversity. Also, based on the family‐level table, a significant ANOSIM analysis of offspring exposed to a bacterial exposure at 18°C (*R* = 0.104, *p* = 0.002) revealed that the four parental vaccination groups (Mat_C_Pat_C_, Mat_C_Pat_V_, Mat_V_Pat_C_, Mat_V_Pat_V_; all BE_V_ and Temp_18_) differ in their dissimilarity from the background [although one group (Mat_V_Pat_C_) showed higher average dissimilarity as the background—a pattern we cannot explain]. In contrast, without bacterial exposure, groups did not differ, even with parental vaccination (*R* = 0.0105, *p* = 0.276; BE_C_ and Temp_18_).

**TABLE 2:** Statistical summary of the selected linear mixed effects model analyzing microbiome diversity. The full model included the Shannon diversity as the dependent variable and Mat, Pat, exp. *Vibrio* log(count+1), and Temp, as well as all interactions as independent variables and Fam as the random effect. *p < 0.05; **p < 0.01; ***p < 0.001.

Variable	Estimate	*χ* ^2^	df	Pr(> *χ* ^2^)
(Intercept)	3.91	5228.76	1	< 0.0001***
Pat_V_	−0.12	2.554	1	0.11
exp. *Vibrio* log(counts+1)	−0.02	10.94	1	0.0009***
Temp_25_	0.12	13.08	1	0.0003***
Pat_V_ ×exp. Vib. log(counts+1)	0.02	6.89	1	0.0087**

**FIGURE 10:**
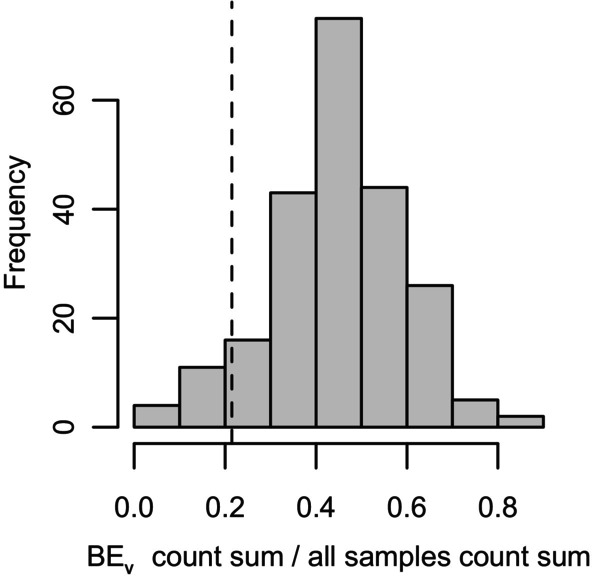
Histogram of proportions of count sums found in bacterial exposure samples per genus. The proportions across genera are normally distributed, and the dashed line indicates the proportion found in the genus *Vibrio* after subtracting the experimental *Vibrio* strain.

As expected, analyses indicated that, when summed up per individual, counts belonging to *Vibrio*‐genus strains (incl. the experimental strain) are more abundant in BE_V_ samples compared to BE_C_ samples (Wilcoxon Test: *W* = 13,924, *n* = 153,154, *p* = 0.006). However, if counts of the experimental *Vibrio* are removed, the BE_C_ samples have considerably more *Vibrio* counts although equal count sums might have been expected (Wilcoxon Test: *W* = 5014, *n* = 153,154, *p* < 0.001). We determined that the proportion of remaining counts found in BE_V_ samples was significantly lower in bacterial strains belonging to the genus *Vibrio* than the average among other genera (one‐sample Wilcoxon signed rank test: *V* = 25,132, *n* = 226, *p*‐value < 0.0001; after normality of the ratio distribution among other genera could not be confirmed: Shapiro–Wilk: *W* = 0.98643, *p*‐value = 0.03; Figure 10), suggesting that the effect of non‐experimental *Vibrio* strains being underrepresented in the BE_V_ samples is likely, at least partially, due to the experimental strain suppressing or outcompeting other *Vibrio* strains, which may occupy similar niches.

## 4 Discussion

Adaptive transgenerational plasticity prepares offspring for the specific environmental settings experienced by their parents. It is predicted to increase their survival in a matching environmental abiotic (e.g., temperature and salinity) and/ or biotic (e.g., microbial assemblage) condition across generations. Here, we investigated TGIP in the pipefish *S. typhle* with their unique evolution of male pregnancy, permitting ample opportunities for parent‐specific transfer of immunological experience through the maternal (eggs), as well as the paternal (male pregnancy) lineage (Beemelmanns and Roth 2017; Roth, Klein, et al. 2012). Specifically, we evaluated how TGIP upon a parent‐specific exposure to heat‐killed bacteria affected the transcriptome‐wide immunological reaction in offspring. Additionally, we tested if parental priming affected the bacteria's abundance in offspring when exposed to the same bacterium, and also how priming and experimental bacteria exposure influenced the offspring microbiota. Finally, this was done in a normal vs. a heat stress environment—an environmental condition expected to be increasingly frequent in the marine realm (Rahmstorf and Coumou 2011).

Offspring temperature treatment simulating a heat stress environment of 25°C had a profound effect on gene expression (Figure 6): enriched GO terms were associated with metabolism and catabolism, as well as the complement system (Makrinos and Bowden 2016). We also identified several heat shock proteins that responded to our heat treatment (*p* < 0.05), including downregulation of *hspb7*, upregulation of *hspb1* and *hspa1*, and a trend for *hsp90ab1* (*p* = 0.054), suggesting that the heat stress made maintaining homeostasis more costly [for comparison, no *hsp*‐gene changed expression in response to the bacterial exposure (Tomanek 2010)]. The heat stress treatment in this study (25°C, when compared to an ambient temperature 18°C) was applied to investigate how the potentially adaptive effect of TGIP on offspring gene expression and microbial community changes under the influence of another stressor. Therefore, gene expression patterns at 18°C are discussed first.

The offspring exposure to orally supplied *Vibrio* bacteria induced a substantial change in gene expression at the control temperature of 18°C, which becomes apparent when inspecting the numbers of significant pairwise comparisons of gene expression (Figure 6). While GO enrichment was not indicative of any specific group of genes contributing to this response, we found that DEGs associated with the bacterial exposure (Figure 6A) were significantly associated with immune cells and specifically more with those of the innate immune system compared to the background (Figure 7A,B). This suggests that the overall response of *S. typhle* offspring to the experimental *Vibrio* challenge included modulation of their immunological activity, especially the innate branch, possibly as the adaptive immune system is still subject to maturation, as was suggested for other juvenile teleosts (Dios et al. 2010; Magnadóttir 2006; Zapata et al. 2006). Non‐exposed juveniles showed significantly lower but still non‐zero abundance of the experimental *Vibrio*, which likely reflects naturally occurring closely related *Vibrio* strains present in all individuals that cannot be differentiated from the experimental strain using the V3–V4 region of their 16S rRNA.

Maternal and paternal immune priming affected the offspring's transcriptomic response to exposure to the experimental *Vibrio* bacteria (Figure 7A), which is supported by lower relative *Vibrio* abundance in guts (Table 1; Figure 5), arguing that TGIP‐related transcriptomic changes indeed facilitated a stronger or more targeted immune response, which supports our hypothesis 1. However, when offspring were left naive, no influence of parental immunological exposure on offspring gene expression was identified. This suggests a bacteria strain‐specific effect of the parental *V. aestuarianus* vaccination that only became effective in a matching parent–offspring bacterial environment, that is, when offspring were exposed to the same bacteria as the parents were vaccinated with. Such specificity argues for a generally low cost of TGIP in non‐matching microbial environments, contradicting our hypothesis 2 (Beemelmanns and Roth 2016a). Additionally, the lack of a transcriptomic response in primed but not bacterial‐exposed juveniles argues for high specificity of the parental immune priming, as suggested in hypothesis 3. However, parental immune priming seemingly also altered the remaining gut microbial community of juvenile pipefish (Table 2): Our ANOSIM suggested that parental immune priming affected microbiota composition. However, our data also showed that the relative abundance of the experimental *Vibrio* affected the composition of the remaining microbiome heterogeneously (Figure 10), and reduced experimental *Vibrio* prevalence (also due to parental immune priming) facilitated a more diverse microbiota in pipefish offspring [Shannon diversity index; higher microbial diversity is generally suggested to be associated with a better health and an improved immune response (Perry et al. 2020)]. Thus, we cannot delineate if the microbiome of parentally primed offspring is different to controls because parental priming also affected non‐focal microbial abundances via direct non‐specific immune responses, or if these shifts in microbiome composition are merely a result of reduced experimental *Vibrio* abundance due to parental priming. Given that juveniles not exposed to bacteria did not differ in their microbiota composition (despite TGIP), we conclude potential support for hypothesis 3.

The effect of maternal and paternal immune priming differed markedly: relatively few DEGs overlapped between the bacterial‐exposed maternal and paternal vaccinated groups and a large number of DEGs was associated with the interaction of the parental effects (Table 1; Figure 6B). Additionally, biparentally vaccinated juveniles were not significantly different in experimental *Vibrio* abundance from juveniles with unvaccinated parents (*p* = 0.076). This suggests that maternal and paternal TGIP effects are not additive but rather complexly interact, similar to what has been found before in this pipefish species using a candidate gene approach during which different gene categories were affected differently by maternal and paternal priming (Roth, Klein, et al. 2012) supporting our hypothesis 4. While GO‐term enrichment analysis did not provide meaningful insights into how parental effects may differ, our immune cell association analysis suggests that DEGs of both groups with a single vaccinated parent are significantly more associated with immune cells than expected by chance. DEGs of the paternal effect are specifically associated with cells of the innate immune response, while maternal effects are associated equally with both innate and adaptive immune cells (relative to the background; Figure 7E–H). We thus conclude that parental TGIP meaningfully affects pathogen defense in *S. typhle*, but the paternal TGIP contribution more strongly involves specifically the innate immune response, which is not the case for the maternal side.

Previous studies on candidate genes have found that maternal and paternal priming tended to affect the adaptive and innate immune branches stronger, respectively, which is in line with our results (Beemelmanns and Roth 2016b; Roth, Klein, et al. 2012). The involvement of the innate immune branch and acquired immunity, possibly via trained innate immunity, could explain how TGIP functions despite the loss of MHCII and other central immune genes of the adaptive immune branch in *Syngnathus* and other syngnathids (Roth et al. 2020). For instance, “trained” epigenetic patterns could be inherited from both parents, but fathers further may have the opportunity to train their developing offspring's immune systems already in the brooding pouch by thus far unknown mechanisms. We also identified several *hsp*‐genes associated with maternal priming and bacterial exposure (*hsp90aa1*, *hspa4b*, *hspb8*, and *hspa1b*, all *p* < 0.05), while none was found to be associated with the paternal priming under bacterial exposure. Together with our immune cell association results, this suggests that maternal priming led to a stronger, but more generic stress response, while paternal priming is more specific and more targeted via a modulation of the innate immune branch, possibly suggesting that “trained immunity” may play a pivotal role in syngnathids' immune biology, as suggested before (Parker et al. 2024). However, the analysis of experimental *Vibrio* prevalence suggests no significant difference in maternal vs. paternal vs. biparental immune priming effects. The involvement of different immunological pathways in the offspring upon maternal vs. paternal immune priming thus seems to facilitate a similar protective effect when it comes to an exposure of the bacteria previously encountered by the parental generation. Thus, parental immune priming in the pipefish *S. typhle* against *V. aestuarianus* appears to be a highly specific and, thus, likely cost‐effective mechanism to boost offspring immune defenses in matching parent‐offspring environments. However, studies showing similar TGIP patterns using more varied microbes (for vaccination and offspring exposure) are necessary to truly shed light on TGIP specificity patterns in *S. typhle*.

TGIP patterns in matching biotic parent–offspring environments were virtually absent in the heat stress treatment. On the transcriptome level, the increased temperature led to a substantial shift in gene expression (Figure 6; Table 1), and associated DEGs were significantly associated with immune cells of both immune system branches. However, at 25°C, only a single gene was identified to respond to the offspring's bacterial exposure treatment, expression changes of only 80 genes could be linked to maternal priming when individuals were also exposed to bacteria, and expression of not a single gene could be linked to paternal or biparental priming when offspring was challenged (Figure 6). Furthermore, maternal priming DEGs were significantly less associated with immune cells than expected by chance, indicating that the maintained response may not be immune‐related.

We considered three scenarios that might have led to these divergent patterns between temperature treatments: (i) the immune response might have been substantially less pronounced at heat stress conditions. As transcriptomic responses are likely linked to energetic costs, responding to external stressors would present a resource allocation trade‐off scenario supporting hypothesis 5. Under severe heat stress, as applied in this experiment, available energy might have predominantly been allocated to the heat response to maintain metabolic homeostasis, hampering the protective effect of TGIP to the bacteria exposure (McKinstry et al. 2017). (ii) Alternatively, the less pronounced immune response reflects the presence of fewer *Vibrio* bacteria. For instance, environmental conditions could have been less favorable for infection with the experimental *Vibrio* strain (relative to other microbes) at high temperatures (25°C) when compared to the 18°C temperature treatment (Table 1; Figure 8; Sheikh et al. 2022). However, the temperature and bacterial exposure treatments were only conducted for 1 day, limiting the effect reduced growth rates might have at 25°C. More importantly, *V. aesturianus* growth in in vitro cultures at 25°C outperforms growth at 18°C (results not shown), entailing that the lower *Vibrio* prevalence in offspring gut identified at 25°C is unlikely to be caused by reduced *Vibrio* growth in pipefish guts upon exposure. (iii) Finally, the transcriptomic responses to heat stress and pathogens may overlap to some extent in juvenile *S. typhle*: our analysis suggests that genes responding to heat stress were over‐proportionally associated with immune cells and thus this broad stress response may have already included most genes of a bacterium‐induced response. Such an effect might also explain why experimental *Vibrio* prevalence overall was lower in the heat treatment compared to the normal temperature group, as was shown by a recent study in which 90% of the genes responding to a *Vibrio alginolyticus* bacterial exposure in Herring (*Clupea harengus*) larvae had also responded in the same direction to a heat stress treatment (Franke et al. 2024). However, in the present study, only 27 DEGs overlap between the pairwise comparisons of Mat_C_Pat_C_BE_C_Temp_18_ vs. Mat_C_Pat_C_BE_C_Temp_25_ (827 DEGs) and Mat_C_Pat_C_BE_C_Temp_18_ vs. Mat_C_Pat_C_BE_V_Temp_18_ (374 DEGs). This small number of shared DEGs does not support the hypothesis that the heat stress and pathogen stress response are redundant.

In conclusion, we believe that the overall reduced relative experimental *Vibrio* prevalence is likely a result of increased abundance of other microbes that benefit more from the increased water temperature, potentially leading to a relatively smaller number of experimental *Vibrio* counts in the sequenced DNA. We therefore believe that the first explanation (i) is most likely, which raises concerns on fish larval immunological vigilance under heat stress scenarios that are expected to increase in frequency and severity over the next decades (Rahmstorf and Coumou 2011). Our data suggests that heat stress does not only lead to a severe change in transcriptomic profiles but also that immunological vigilance can be compromised likely due to a resource allocation trade‐off, as has been discussed before for other environmental stressors and which supports hypothesis 5 (Birrer, Reusch, and Roth 2012; Scharsack and Franke 2022). Thus, increases in environmental temperature regimes may threaten this pipefish's natural populations as efficient immune priming may not be possible anymore, hampering the resilience toward relatively sudden shifts in microbial compositions expected in their environment.

For host organisms, TGIP can mediate some of the challenges arising from an environmental microbiome composition changing too fast for genetic adaptations to occur. Vaccinations with heat‐killed bacteria in parents, simulating pathogen exposure, can induce effective and specific immune priming in offspring, with mothers and fathers affecting the offspring's immune responses differently, protecting from pathogens already encountered in the parental generation. This adaptive transgenerational plasticity seems highly cost‐effective in a situation of non‐matching biotic environments. However, heatwaves, which are expected to increase in frequency due to climate change, have the potential to negate TGIP benefits, possibly via a resource allocation trade‐off. This raises substantial concerns on how animals will cope with climate change‐induced shifts in pathogen assemblages, ultimately predicting alarming scenarios of emerging marine disease.

## Author Contributions

**Ralf F. Schneider:** conceptualization (equal), data curation (equal), formal analysis (lead), investigation (equal), methodology (equal), resources (supporting), visualization (lead), writing – original draft (equal), writing – review and editing (equal). **Arseny Dubin:** data curation (equal), formal analysis (supporting), methodology (supporting), writing – original draft (supporting), writing – review and editing (supporting). **Silke‐Mareike Marten:** data curation (supporting), methodology (supporting), writing – original draft (supporting). **Olivia Roth:** conceptualization (lead), data curation (supporting), funding acquisition (lead), investigation (lead), methodology (equal), project administration (lead), writing – original draft (equal), writing – review and editing (equal).

## Ethics Statement

All experiments were conducted in accordance with local ethics regulations (University of Kiel Antrag §7 V242‐35168/2018).

## Conflicts of Interest

The authors declare no conflicts of interest.

## Data Availability

Raw reads of the transcriptomic and microbiome data sets are available on NCBI (project ID PRJNA1173975). All processed data are available on Dryad (https://doi.org/10.5061/dryad.5qfttdzgq).
